# A culturally grounded Arabic naming task differentiates Saudi Arabic-speaking adults with post-stroke aphasia from neurotypical adults

**DOI:** 10.3389/fneur.2026.1954019

**Published:** 2026-09-16

**Authors:** Maha Aldera, Mohammed AlHarbi, Abrar Alduraibi, Majd Almeshal, Banan Aldosari, Raghad Baruqan, Taif Alsubaie, Nisreen Al Awaji

**Affiliations:** 1 Department of Health Communication Sciences, College of Health and Rehabilitation Sciences, Princess Nourah bint Abdulrahman University, Riyadh, Saudi Arabia; 2 Department of Audiology and Speech-Language Pathology, College of Medical Rehabilitation Sciences, Taibah University, Medina, Saudi Arabia; 3 King Fahad Medical City, Riyadh, Saudi Arabia

**Keywords:** anomia, aphasia, Arabic naming, confrontation naming, neurorehabilitation, post-stroke aphasia, Saudi Arabia, speech-language pathology

## Abstract

**Background/objectives:**

Anomia is a common linguistic consequence of post-stroke aphasia. Widely used confrontation-naming measures were developed primarily in English-speaking populations, and item familiarity may not transfer to Arabic speakers. We developed the Arabic Naming Test (ANT) as a culturally grounded picture-naming task and examined its preliminary clinical feasibility in Saudi Arabic-speaking adults.

**Methods:**

In this cross-sectional convenience-sample study, 75 neurotypical native Arabic-speaking adults and 30 adults with post-stroke aphasia completed a 90-item picture-confrontation naming task. Accuracy and response latency were recorded; participants with aphasia also completed a sequential semantic–phonemic cueing protocol. Welch tests and age-adjusted sensitivity analyses compared groups.

**Results:**

The aphasia group had lower ANT scores than neurotypical peers (*M* = 49.50, SD = 31.66 vs. *M* = 87.55, SD = 3.48; Welch’s *t*(29.3) = −6.57, *p* < 0.001; Hedges’ *g* = −2.21) and slower mean response latency per item (*M* = 1.80 s vs. *M* = 1.06 s; Welch’s t(29.8) = 3.77, *p* < 0.001; Hedges’ *g* = 1.24). These group differences remained statistically significant in age-adjusted sensitivity analyses. Sequential semantic–phonemic cueing analyses of 30 participants showed reductions in description, miscellaneous, no-response, and semantic errors after adjustment for multiple comparisons. On average, 17.17 items (SD = 18.46) were named correctly after phonemic cueing and 2.90 items (SD = 3.32) after semantic cueing alone.

**Conclusion:**

In this preliminary convenience sample, the ANT showed substantial known-groups differences between Saudi Arabic-speaking neurotypical adults and adults with post-stroke aphasia and generated descriptive cueing and error-profile observations. These findings support continued development of a culturally grounded Arabic naming measure; however, larger, demographically balanced multi-site studies are needed before formal reliability, psychometric validation, diagnostic accuracy, or clinically meaningful thresholds can be established.

## 1 Introduction

Stroke is the second leading cause of death and a leading cause of long-term adult disability worldwide. The World Stroke Organization estimates that the lifetime risk of stroke has risen by roughly 50% over the past two decades, such that approximately one in four adults may experience a stroke (1). Saudi Arabia is projected to see a particularly steep rise, with modeling suggesting first-ever stroke incidence could increase by 57–67% over coming decades as the population ages (2). As stroke survival has improved even as incidence climbs, the number of Saudi adults living with post-stroke communication disorders is expected to grow substantially, increasing demand for locally valid assessment tools.

Aphasia, an acquired language disorder most commonly resulting from left-hemisphere stroke, affects far more than communication in the narrow sense. It disrupts family roles, employment, and social participation, and is consistently associated with reduced community integration and quality of life relative to stroke survivors without aphasia (3). Among its component impairments, anomia, the inability to retrieve familiar words on demand, is considered the most pervasive and diagnostically central feature of aphasia, present across virtually all clinical subtypes and often the last symptom to resolve (4). For this reason, confrontation naming tasks occupy a central place in aphasia assessment: they are used to establish diagnosis, characterize the profile and severity of word-finding impairment, and track change over the course of treatment.

The Boston Naming Test (BNT) (5, 6) and the Philadelphia Naming Test (PNT) (7) are the most widely used confrontation naming measures internationally. Both ask examinees to name line drawings or photographs under standardized timing and cueing conditions, yielding scores that are well characterized in English-speaking, predominantly North American normative samples (8). However, naming accuracy is strongly influenced by an item’s cultural and linguistic familiarity rather than by word-finding ability alone, which limits the cross-cultural portability of any naming instrument developed in one linguistic community and applied in another. This concern has motivated formal re-standardization of the BNT in multiple countries: the Turkish adaptation replaced or added 29 items to reflect locally familiar objects (9), the Korean version added 50 new items (10), and the Portuguese adaptation added 20 items (11), each substituting or supplementing items following field testing with native speakers. Even within English-speaking contexts, the BNT has required adaptation in Australia and New Zealand to remove or replace items unfamiliar to those populations (12), underscoring that item familiarity, not just language, drives cross-cultural test performance. Across these adaptations, naming performance has also been consistently linked to age and educational attainment, with less consistent evidence for an effect of gender (9–11).

The need for an Arabic-specific naming approach is also linguistic, not solely cultural. Arabic is characterized by diglossia: Modern Standard Arabic is used primarily in formal, written, and educational contexts, whereas spoken regional varieties are acquired naturally and used in everyday communication. Arabic dialects may differ at lexical, phonological, morphological, and syntactic levels; consequently, a pictured referent may legitimately elicit different spoken labels or forms across communities. In confrontation naming, scoring that accepts only a single Standard Arabic label could therefore confound dialectal lexical variation with impaired word retrieval. Arabic also has a rich non-linear root-and-pattern morphological system, in which consonantal roots and vocalic patterns jointly encode lexical and grammatical information. Although the ANT was not designed to isolate morphological processing, these linguistic characteristics further limit the validity of direct translation from English-language naming instruments and support the use of a Saudi Arabic item set and scoring rules that explicitly define acceptable lexical variants (13, 14).

Arabic is one of the world’s most widely spoken languages, used daily by more than 400 million people, and encompasses linguistically diverse communities and regional dialects that may influence lexical familiarity and naming performance (15). A recent systematic review concluded that Arabic aphasia research remains comparatively underdeveloped and noted the continuing reliance in some settings on informal assessments or translated Western-language measures that may not adequately capture Arabic linguistic and cultural features (13). The review further highlighted dialectal variation as an important consideration in the development of clinically useful assessment materials (13). Broader neurogenic-language assessment literature likewise emphasizes that linguistically fair assessment should account for the psycholinguistic, sociolinguistic, and cultural properties of the target language and its dialects, rather than rely on literal translation of instruments developed in other language communities (16, 17).

Arabic-language assessment development has nevertheless advanced in important ways. In Saudi Arabic, Alyahya and Druks (18) adapted the Object and Action Naming Battery and reported item-level name-agreement data together with spoken-word frequency, imageability, age of acquisition, and visual-complexity measures. In Gulf Arabic, Altaib et al. (19) developed and preliminarily evaluated the Short Aphasia Test for Gulf Arabic speakers through staged cultural-linguistic review. These studies demonstrate the value of culturally and linguistically responsive development rather than direct translation of English-language tests. Building on this emerging literature and on evidence that direct application of the Boston Naming Test can yield item-familiarity mismatches for Saudi Arabic speakers (20), the present study examines the preliminary clinical feasibility of a 90-item culturally grounded Arabic picture-naming task developed for Saudi Arabic-speaking adults.

The ANT was developed from two complementary foundations. Its picture-confrontation naming format was informed by the Boston Naming Test and Philadelphia Naming Test, and its target pool was drawn from Arabic word materials developed by Aldera for research on acquired dyslexia and dysgraphia in Arabic-speaking adults with aphasia. Those materials were developed within a dual-route language-processing framework and considered lexical variables relevant to Arabic processing (21). For the preliminary ANT, 90 culturally relevant targets varying in difficulty according to frequency of use were selected, and original photographs were reviewed for clarity and target-picture correspondence. As a prespecified final-item selection algorithm and item-level lexical metrics were not retained, the present study does not claim that the 90 items were formally matched or balanced on these variables.

The present study examined the preliminary clinical feasibility of the ANT in neurotypical Saudi adults and adults with post-stroke aphasia. Specifically, this study aimed to: (1) describe naming performance in the present neurotypical convenience sample; (2) examine whether the ANT task differentiates the present aphasia and neurotypical samples and characterize the observed naming-error patterns; and (3) describe response to a sequential semantic–phonemic cueing hierarchy as preliminary information to guide future clinical and therapeutic research, rather than as evidence of treatment-monitoring responsiveness.

## 2 Materials and methods

### 2.1 Design and participants

This cross-sectional, between-subjects study compared naming performance in neurotypical adults and adults with aphasia. Participants were recruited via convenience sampling in Riyadh, Saudi Arabia, between May 2024 and February 2026. As a preliminary feasibility study, the sampling strategy was intended to support initial clinical comparison rather than to generate population-representative norms or adequately balanced subgroup samples. The neurotypical group comprised 75 native Arabic-speaking adults over 18 years of age with no history of neurological, psychiatric, or language disorder. The aphasia group comprised 30 adults with a confirmed clinical diagnosis of aphasia secondary to stroke, established by a certified speech-language pathologist. Available study data for the aphasia group included age, handedness, clinician-recorded aphasia descriptors, and stroke information where documented. The clinician- recorded aphasia descriptors included language profile and a clinical severity label; these descriptors were used for descriptive purposes only and were not standardized classification or severity scores. Time since stroke, the specific assessment or diagnostic battery used to establish aphasia, standardized aphasia-severity scores, and complete stroke lesion/etiology data were not collected prospectively and cannot be retrospectively obtained or verified. Exclusion criteria were non-native Arabic speaker status, age under 18 years, and neurological or psychiatric conditions unrelated to the presenting stroke that could affect language performance.

### 2.2 Ethical considerations

The study protocol was approved by the Institutional Review Board of Princess Nourah bint Abdulrahman University and by the Institutional Review Board of King Fahad Medical City (approval nos. 23-0761 and 23-695, respectively). All participants provided written informed consent prior to enrollment. Data were de-identified, and access was restricted to the principal investigator.

### 2.3 Test materials

The ANT is a 90-item picture-confrontation naming task developed for Saudi Arabic-speaking adults. Its development was informed by the Boston Naming Test and Philadelphia Naming Test frameworks, while the target words were selected from an earlier Arabic word list developed by Aldera (21). The earlier source materials considered lexical variables relevant to Arabic processing, including word frequency, imageability/concreteness, word length, grammatical class, morphology, regularity, and lexicality. The documented selection rationale for the preliminary ANT was to retain words related to Arabic culture and to include items that ranged in difficulty according to frequency of use. Original real-world photographic stimuli were developed to represent the selected words. Six licensed speech-language pathologists reviewed the photographs for clarity, size, and correspondence between each image and its intended target word. The final stimuli were presented electronically as a timed PowerPoint sequence on a tablet screen at a viewing distance of approximately 20 cm, with each image displayed for 20 s and accompanied by an audible onset signal used to support response-latency timing. A parallel scoring booklet was used by examiners to record responses in real time. The development documentation did not include a prespecified item-selection algorithm, formal inclusion/exclusion criteria beyond cultural relevance and frequency-related variation, or final ANT item-level metrics for word frequency, familiarity, imageability, age of acquisition, visual complexity, word length, grammatical category, or name agreement. Accordingly, the present study does not claim that items were formally matched, standardized, or balanced on these psycholinguistic properties. The expert picture review was intended to support preliminary content appropriateness of the stimuli; it did not establish formal item-level psychometric validation of the final ANT.

### 2.4 Procedure

Four trained examiners administered the ANT using standardized instructions. Participants were asked to produce a single-word name for each pictured item as soon as it appeared, and all responses were audio-recorded. Neurotypical participants completed one administration; responses were scored as correct or classified into one of nine predefined error categories: nonword phonological (N), real-word phonological (F), semantic (S), description (D), miscellaneous (Misc), mixed (M), unrelated (U), perseveration (P), or no response (NR). Participants with aphasia completed an initial uncued naming trial. For items not named spontaneously within the uncued response window, a fixed sequential cueing hierarchy was applied. Semantic cues consisted of a standardized descriptive prompt referring to the category, function, or a salient associative feature of the target item. Phonemic cues consisted of the initial phoneme or syllable of the target word, presented in isolation without additional co-articulatory information. Both semantic and phonemic cues were delivered verbally, live by the examiner, using pre-specified wording that was held constant across examiners and participants. The cues were not presented as written text within the PowerPoint and were not delivered as pre-recorded audio. The semantic cue was delivered immediately after the uncued response window for items that were not named spontaneously. When a response remained incorrect after the semantic-cue stage, the phonemic cue was delivered immediately thereafter. As semantic cues preceded phonemic cues, the protocol was not designed to compare the independent effects of the two cue types. Accordingly, cueing outcomes were interpreted descriptively as responses to a fixed sequential hierarchy and were not used to estimate the independent clinical utility or relative effectiveness of semantic and phonemic cues. Response latency, timed from stimulus onset to response onset, was measured using Audacity software (version 3.5.1).

### 2.5 Scoring and exclusion criteria

Regional or dialectal lexical variants that unambiguously referred to the target concept were scored as correct. Trials with no response were coded as incorrect/no response and were excluded from response-latency analyses. Recordings that were too noisy or unintelligible to permit reliable scoring were excluded from the relevant response-latency or error-type analyses. Trials with missing response-latency values, for example because of technical error, were excluded from latency analyses but retained for accuracy analyses when a codable response was available. For the sequential cueing analyses, error types were coded at two distinct stages. “Before cueing” referred to the error classification assigned to the initial uncued naming response. “After cueing” referred to the error classification assigned to the final response after the applicable sequential cueing process had ended: an item resolved after the semantic cue was recorded as correct after semantic cueing, whereas an item unresolved after semantic cueing proceeded to phonemic cueing and, if named correctly, was recorded as correct after phonemic cueing. Correct responses after cueing were therefore tabulated separately and were not included in the post-cue error categories. If a final post-cue response remained incorrect, it was classified using the same error taxonomy as the initial response. Consequently, an item could receive a different error classification after cueing than at the uncued stage.

### 2.6 Statistical analysis

Data were analyzed using SPSS version 25.0. Demographic characteristics were summarized using frequencies and percentages. Chi-square tests were used for exploratory categorical group comparisons, with cautious interpretation where expected cell counts were small. Welch independent-samples t-tests were used to compare ANT score and response-latency outcomes between groups and between younger and older subgroups because they do not assume equal variances. Hedges’ g was calculated for the primary between-group comparisons. Age-adjusted linear regression models included group and age as predictors and used robust standard errors as sensitivity analyses for the primary group comparisons. Among participants with aphasia, paired-samples t-tests compared error frequencies before and after the sequential cueing hierarchy. The Holm procedure was used to adjust *p*-values across the nine exploratory error-category comparisons. Education, age-subgroup, and aphasia-profile analyses were treated as exploratory because the convenience sample produced sparse and uneven category sizes. These analyses were not intended to provide stable subgroup estimates or population-level normative inferences. All tests were two-sided, and *p* < 0.05 was considered statistically significant.

## 3 Results

### 3.1 Sample characteristics

The neurotypical group (*N* = 75) and aphasia group (*N* = 30) did not differ significantly in gender distribution (*χ*^2^ = 2.97, *p* = 0.085) or nationality (*χ*^2^ = 0.34, *p* = 0.562). The aphasia group included a higher proportion of participants aged 41 years or older than the neurotypical group (76.7% vs. 46.7%; *χ*^2^ = 7.80, *p* = 0.005). Education data were available for 74 neurotypical participants and all 30 participants with aphasia. In the collapsed education comparison, the groups did not differ significantly (*χ*^2^ = 2.07, *p* = 0.558); this finding should be interpreted cautiously because of small, expected cell counts (Table 1). These demographic patterns reflect the convenience-sampling design and should not be interpreted as representative of the broader Saudi Arabic-speaking population with or without aphasia.

**Table 1:** Demographic characteristics of the neurotypical and aphasia groups.

Variable	Category	Neurotypical (*N* = 75) *n* (%)	Aphasia (*N* = 30) *n* (%)	*χ* ^2^	*p*
Gender	Male	24 (32.0)	15 (50.0)	2.97	0.085
	Female	51 (68.0)	15 (50.0)		
Nationality	Saudi	72 (96.0)	28 (93.3)	0.34	0.562
	Non-Saudi	3 (4.0)	2 (6.7)		
Age group	Younger (≤40y)	40 (53.3)	7 (23.3)	7.80**	0.005
	Older (41y+)	35 (46.7)	23 (76.7)		
Education	Illiterate–Middle school	11 (14.9)	8 (26.7)	2.07	0.558
	High school–Diploma	18 (24.3)	7 (23.3)		
	College–Bachelor’s	39 (52.7)	13 (43.3)		
	Master’s–PhD	6 (8.1)	2 (6.7)		

*p* < 0.01. Education data were available for 74/75 neurotypical participants and 30/30 participants with aphasia; education percentages use these available-data denominators. Nationality and education chi-square results should be interpreted descriptively because of small expected cell counts.

Table 2 presents the available clinical characteristics of the 30 participants with aphasia. Participants with aphasia ranged in age from 20 to 73 years (*M* = 49.77, SD = 15.08), and 29 participants (96.7%) were right-handed. The recorded aphasia descriptors classified 14 participants (46.7%) as having expressive non-fluent aphasia, 11 (36.7%) as having expressive fluent aphasia, and 5 (16.7%) as having mixed non-fluent aphasia. Recorded clinical severity labels were mild or mild-to-moderate for 12 participants (40.0%), moderate for 9 (30.0%), moderately severe or moderate-to-severe for 3 (10.0%), and severe for 6 (20.0%). Stroke was documented for all participants; however, type and/or site were not recorded for 21 participants (70.0%). Time since stroke, standardized aphasia-severity scores, and the specific diagnostic assessment were not collected prospectively and cannot be retrospectively obtained or verified.

**Table 2:** Clinical characteristics of participants with aphasia (*N* = 30).

Characteristic	*n* (%) or summary
Age, years	*M* = 49.77, SD = 15.08; range = 20–73
Right-handed	29 (96.7)
Left-handed	1 (3.3)
Recorded aphasia language profile	
Expressive non-fluent	14 (46.7)
Expressive fluent	11 (36.7)
Mixed non-fluent	5 (16.7)
Recorded clinical severity label	
Mild or mild-to-moderate	12 (40.0)
Moderate	9 (30.0)
Moderately severe or moderate-to-severe	3 (10.0)
Severe	6 (20.0)
Recorded stroke information	
Stroke documented; type/site not recorded	21 (70.0)
Left middle cerebral artery stroke	3 (10.0)
Other recorded type/site*	6 (20.0)

Recorded aphasia language-profile and clinical-severity labels were descriptive, not standardized classifications or validated severity scores. Stroke type/site information was incomplete; therefore, no subtype- or lesion-specific analyses were conducted. Time since stroke, the diagnostic assessment or battery, and standardized severity scores were not prospectively collected. *Other recorded type/site refers to stroke types and/or sites documented in the available clinical records other than left middle cerebral artery stroke.

### 3.2 Known-groups differences: neurotypical vs. aphasia

Naming performance differed markedly between the neurotypical and aphasia groups (Table 3). Compared with neurotypical participants, participants with aphasia had substantially lower ANT scores and longer total and mean response latencies. Welch tests were used because outcome variances differed between groups, particularly for ANT score and latency outcomes. All three primary group differences remained statistically significant in age-adjusted robust sensitivity analyses, providing preliminary known-groups evidence in this convenience sample. Because the aphasia group was older on average, these findings should be interpreted cautiously and examined further in future age-balanced studies.

**Table 3:** Welch independent-samples comparisons of neurotypical and aphasia groups.

Variable	Group	*N*	*M*	SD	Welch t	df	*p*
Age (years)	Neurotypical	75	40.44	15.28	−2.85	54.1	0.006
	Aphasia	30	49.77	15.08			
Score (/90)	Neurotypical	75	87.55	3.48	6.57	29.3	<0.001
	Aphasia	30	49.50	31.66			
Total response time (s)	Neurotypical	75	94.56	17.57	−3.50	29.9	0.001
	Aphasia	30	152.68	90.17			
Mean latency/item (s)	Neurotypical	75	1.06	0.20	−3.77	29.8	<0.001
	Aphasia	30	1.80	1.07			

Welch tests were used. Absolute Hedges’ g values were 2.21 for ANT score, 1.15 for total response time, and 1.24 for mean latency per item; directions of the group differences are shown by the group means. Primary group differences remained statistically significant in age-adjusted robust sensitivity analyses (all *p* < 0.01).

### 3.3 Exploratory age-subgroup analyses

Exploratory age-subgroup analyses showed no statistically significant differences in ANT score, total response time, or mean latency per item between younger and older neurotypical participants (Table 4). Within the aphasia group, age was likewise not significantly associated with ANT score, total response time, or mean latency per item (Table 5). The younger aphasia subgroup was small (*n* = 7); therefore, these analyses have limited precision and do not provide definitive age-stratified normative evidence.

**Table 4:** Welch independent-samples comparison of younger and older neurotypical adults (*N* = 75).

Variable	Group	*N*	*M*	SD	Welch t	df	*p*
Score (/90)	Younger	40	87.53	3.49	−0.06	71.6	0.955
	Older	35	87.57	3.52			
Total response time (s)	Younger	40	95.43	20.13	0.46	70.3	0.644
	Older	35	93.57	14.34			
Mean latency/item (s)	Younger	40	1.06	0.22	0.13	71.6	0.900
	Older	35	1.06	0.17			

No comparison reached statistical significance. Welch tests were used.

**Table 5:** Welch independent-samples comparison of younger and older adults with aphasia (*N* = 30).

Variable	Group	*N*	*M*	SD	Welch t	df	*p*
Score (/90)	Younger	7	61.57	28.06	1.25	11.3	0.236
	Older	23	45.83	32.35			
Total response time (s)	Younger	7	169.68	56.84	0.75	17.9	0.466
	Older	23	147.51	98.57			
Mean latency/item (s)	Younger	7	1.88	0.63	0.30	19.7	0.766
	Older	23	1.78	1.19			

No comparison reached statistical significance. These subgroup analyses are exploratory because of the small younger aphasia subgroup.

### 3.4 Exploratory education and aphasia-subtype analyses

Education data were available for 74 neurotypical participants. Across the four-category education grouping, the parametric and rank-based analyses produced discordant findings in the context of small and uneven cells. Education-related results were therefore treated as exploratory and do not provide a stable estimate of the association between education and ANT performance. The aphasia sample included small, heterogeneous, and incompletely characterized profile groups. The available language-profile and clinical severity descriptors were not standardized measures, and detailed time-post-stroke, diagnostic-assessment, and lesion/etiology information were not collected prospectively and cannot be retrospectively obtained or verified. Therefore, no subtype-specific conclusions are reported.

### 3.5 Effect of sequential semantic–phonemic cueing

Table 6 distinguishes error classifications at the initial uncued naming attempt from classifications of the final response after the applicable sequential cueing process. Correct responses after semantic cueing and after phonemic cueing were recorded separately and are not included in the final post-cue error totals. The distribution of response types differed between the initial uncued stage and the final post-cue stage among the 30 participants with aphasia who completed the sequential cueing protocol (Table 6). No response was the most frequent outcome before and after cueing, but the total frequency decreased from 593 to 250 instances; semantic errors decreased from 136 to 18 instances. As an item that remained incorrect could receive a different final error classification after cueing, changes in category totals reflect both resolution of errors and shifts from one error type to another; for example, a no-response or semantic error could become a phonological approximation after cueing. Thus, increases in nonword or formal/real-word phonological errors should be interpreted as changes in the form of residual incorrect responses rather than as a deterioration in performance. Before multiplicity adjustment, paired-samples t-tests showed reductions in description, miscellaneous, unrelated, no-response, and semantic errors. After Holm adjustment across nine exploratory error-type comparisons, reductions in description, miscellaneous, no-response, and semantic errors remained statistically significant. Participants produced a mean of 17.17 correct responses (SD = 18.46) after phonemic cueing and 2.90 correct responses (SD = 3.32) after semantic cueing alone. As semantic cues were always presented before phonemic cues, these findings describe response to a fixed sequential cueing hierarchy and do not provide a counterbalanced comparison of the independent effects or relative clinical utility of the two cue types. The results therefore cannot determine whether phonemic cueing is intrinsically more effective than semantic cueing, nor can they identify an optimal cue type for individual clinical decision-making (see Table 7).

**Table 6:** Participant-level frequency of each error type at initial uncued naming and at the final response after sequential semantic–phonemic cueing (*N* = 30).

Error type	Minimum count per participant: uncued	Maximum count per participant: uncued	Total errors: uncued	Minimum count per participant: final post-cue response	Maximum count per participant: final post-cue response	Total errors: final post-cue response
Nonword phonological (N)	0	8	34	0	19	64
Formal/real-word phonological (F)	0	13	21	0	10	26
Description (D)	0	16	64	0	8	13
Miscellaneous (Misc)	0	4	34	0	4	9
Mixed (M)	0	1	7	0	1	5
Unrelated (U)	0	85	269	0	81	136
Perseveration (P)	0	22	55	0	9	11
No response (NR)	0	90	593	0	90	250
Semantic (S)	0	18	136	0	6	18

“Uncued” refers to the initial naming response before any cue was given. “Final post-cue response” refers to the final response after the sequential cueing procedure, in which a semantic cue was provided first and a phonemic cue was provided only when needed. Minimum and maximum values represent the number of items assigned to each error category for an individual participant (range, 0–90); totals are summed across all 30 participants. Correct responses after cueing were recorded separately and are not included in the final post-cue error counts. Remaining incorrect responses were reclassified after cueing and could therefore differ from their initial error category.

**Table 7:** Within-participant paired comparisons of category-specific error counts at initial uncued naming and at the final response after sequential semantic–phonemic cueing (*N* = 30).

Error type	M uncued	M final post-cue response	*t*	df	*p*
Nonword (N)	1.13	2.13	−1.19	29	0.244
Formal/real-word phonological (F)	0.70	0.87	−0.69	29	0.493
Description (D)	2.13	0.43	3.44	29	0.002
Miscellaneous (Misc)	1.13	0.30	4.33	29	<0.001
Mixed (M)	0.23	0.17	0.63	29	0.536
Unrelated (U)	8.97	4.53	2.50	29	0.018
Perseveration (P)	1.83	0.37	1.84	29	0.077
No response (NR)	19.77	8.33	4.02	29	<0.001
Semantic (S)	4.53	0.60	4.82	29	<0.001

Values are unadjusted paired-samples t-test p values. The uncued and final post-cue stages are defined in Table 6. Correct responses after semantic or phonemic cueing were tabulated separately and excluded from final post-cue error counts. Following Holm adjustment across nine exploratory comparisons, description (*p* = 0.011), miscellaneous (*p* = 0.001), no response (*p* = 0.003), and semantic errors (p < 0.001) remained statistically significant; unrelated errors did not (adjusted *p* = 0.092).

### 3.6 Qualitative error patterns

Beyond the quantitative error taxonomy, qualitative review of participants’ responses indicated that lexical variation in Arabic contributed to scoreable response variation. Several participants produced regionally or colloquially acceptable alternatives for target items, such as /ʃɑːɾiʕ/ and /tˤɑːɾɪq/ for “road/street,” or /kuːʕ/ and /mirfaq/ for “elbow.” Other responses appeared to reflect stimulus ambiguity rather than word-finding impairment; for example, a picture intended to depict “boy” was named “picture,” and a water-themed item was named “bottle.” These observations identify items that may warrant refinement in future versions of the ANT.

## 4 Discussion

This study reports an initial clinical feasibility evaluation of the ANT, a culturally grounded picture-naming task developed for Saudi Arabic-speaking adults. It complements emerging Arabic-language assessment research, including the Saudi Arabic adaptation of the Object and Action Naming Battery (18) and the preliminary Gulf Arabic Short Aphasia Test (19), while focusing specifically on a 90-item single-word naming task and sequential cueing observations. As Basura et al. (20) demonstrated, direct application of the BNT to Saudi Arabic speakers yields reduced naming accuracy that may reflect item-familiarity mismatch rather than genuine word-finding impairment. The present findings show that a task built from a culturally grounded Arabic item set differentiated this neurotypical convenience sample from this aphasia convenience sample, supporting further development of a locally grounded assessment approach.

The large score and latency differences between the neurotypical and aphasia groups demonstrate preliminary known-groups differences. Participants with aphasia scored, on average, 38.05 points lower on the 90-item task and required substantially more time per item to respond; these findings remained statistically significant in age-adjusted robust sensitivity analyses. This pattern is consistent with the clinical importance of confrontation naming in aphasia assessment (4). However, the present data do not establish formal psychometric validity, normative reference values, a diagnostic threshold, full standardization, or treatment-monitoring responsiveness; those claims require independent, demographically balanced validation samples and longitudinal evidence. Future ANT development should follow the item-level reporting approaches used in Arabic naming-battery adaptation work by evaluating name agreement, lexical frequency, imageability, age of acquisition, and visual complexity, as well as possible variation across Saudi regions and dialect communities (18).

Near-ceiling neurotypical performance (mean ANT score = 87.55/90) suggests that the culturally familiar item set was readily accessible for many participants in this sample. This finding supports item appropriateness but may also indicate a ceiling effect, which could limit sensitivity to subtle naming difficulty among high-performing individuals. Future normative and item-level analyses should examine item difficulty, discrimination, and differential functioning across age, education, gender, and regional dialect, consistent with core psychometric considerations in aphasia test development (8).

Age-related differences were not demonstrated in these exploratory analyses; however, the present convenience samples were not designed to provide definitive age-stratified normative evidence. Larger, adequately powered age-stratified normative studies are therefore needed to determine the contribution of age to Arabic naming performance.

Education-related findings should also be interpreted cautiously. Education data were missing for one neurotypical participant, and the four-category analysis produced discordant parametric and rank-based findings in the context of sparse, uneven cells. Accordingly, the present study cannot provide a stable estimate of the relationship between education and ANT performance. Future normative work should recruit sufficient participants across education strata and pre-specify whether education is modeled continuously or through clinically meaningful categories.

Gender effects on naming remain unsettled in the broader BNT literature: Welch et al. (22) reported a male advantage on select items, whereas Turkish (9), Korean (10), and Portuguese (11) BNT adaptations found no clear gender effect. The current neurotypical sample was female-predominant (68.0%), and the sample was not designed to test gender effects. This remains an appropriate target for larger, gender-stratified ANT validation studies.

The cueing analyses provide useful preliminary findings. Error patterns differed between the initial uncued stage and the final post-cue stage, with lower frequencies of no-response and semantic errors, as well as in description and miscellaneous errors after multiplicity adjustment. The higher number of items named after a phonemic cue than after a semantic cue is descriptive of this sequential protocol. Because phonemic cues were offered only after semantic cues had not produced a correct response, the data cannot determine whether phonemic cueing is intrinsically more effective than semantic cueing, whether the observed pattern reflects cue type rather than cue order, or which cue type would be most clinically useful for a given individual. A future randomized or counterbalanced design is needed to compare the independent contribution and clinical utility of cue types.

Within research and supervised clinical contexts, the combined total-score, latency, error-profile, and cue-responsiveness observations may help describe an individual’s naming presentation more comprehensively; however, prospective studies are needed to determine whether these measures reliably track response to intervention or support clinical decision-making.

Finally, the qualitative error analysis illustrates a feature of Arabic that naming-test developers in other languages have had to grapple with as well: a single referent may have multiple regionally or registerially acceptable names. Just as BNT adaptations in Turkey, Korea, and Portugal required adding or replacing items to fit local lexical norms (9–11), the acceptable-synonym patterns observed here (e.g., /ʃɑːɾiʕ/ versus /tˤɑːɾɪq/ for “road”) suggest that future ANT scoring guidelines should explicitly define acceptable synonym sets, and that a subset of items generating ambiguous or ambiguously depicted responses may need re-illustration in future test revisions.

### 4.1 Study limitations

Several limitations qualify these findings. First, recruitment was restricted to Riyadh and used convenience sampling, which may introduce selection bias and limit generalizability to other Saudi regions, other Arabic-speaking communities, and the broader population of adults with post-stroke aphasia. Second, the modest sample size, particularly for the aphasia group, limits the precision of descriptive estimates and precludes stable inferences for education, gender, aphasia-profile, or other subgroup comparisons. The primary between-group findings should therefore be interpreted as preliminary known-groups evidence in this convenience sample rather than as population-representative normative or diagnostic evidence. Third, background noise in some clinical recording environments complicated response-time scoring and, in some cases, error-type classification for participants with aphasia. Fourth, some participants with aphasia appeared to experience fatigue during testing, which may have depressed performance independent of underlying language ability. Fifth, convenience sampling produced uneven distributions across age and education levels. In addition, although recorded aphasia profile and clinical severity descriptors, as well as selected stroke information, were available in the study dataset, time since stroke, a standardized aphasia-severity score, the specific assessment or diagnostic battery used to establish aphasia, and complete stroke lesion/etiology information were not collected prospectively and cannot be retrospectively obtained or verified. These omissions limit interpretation of how ANT performance may vary by clinical characteristics. Future validation studies should prospectively collect these variables using prespecified and standardized procedures. In addition, the fixed semantic-then-phonemic cue order confounded cue type with cue sequence. The cueing data therefore cannot establish the independent effect or comparative clinical utility of semantic and phonemic cues, and they should not be used to guide cue-selection decisions.

The final selection of 90 items was guided by cultural relevance and variation in frequency of use; however, the available development documentation did not specify a prespecified item-selection algorithm, formal exclusion criteria, or final-ANT item-level metrics for word frequency, familiarity, imageability, age of acquisition, visual complexity, word length, grammatical category, or name agreement. Although six speech-language pathologists reviewed the photographic stimuli for clarity, size, and target-picture correspondence, this review does not substitute for formal item-level linguistic or psychometric validation. Future ANT development should prospectively document inclusion and exclusion decisions, obtain Arabic normative ratings of familiarity and imageability, quantify word-frequency and word-length distributions, document grammatical categories, and conduct item-level psychometric analyses. Finally, formal inter-rater and test–retest reliability analyses were beyond the scope of the present preliminary study. These measurement properties should be evaluated in future validation studies using prospectively planned paired ratings and repeat administrations.

## 5 Conclusion

This study provides preliminary clinical feasibility and known-groups findings for a culturally grounded Arabic naming task in Saudi Arabic-speaking adults with post-stroke aphasia. In this convenience sample, the ANT showed substantial differences between aphasia and neurotypical groups in naming accuracy and response latency, and it generated descriptive error-profile and sequential cueing observations. These findings support continued development of a locally grounded Arabic assessment approach. Larger, demographically balanced multi-site studies are needed to establish normative reference values, formal reliability, item-level psychometric properties, diagnostic accuracy, and clinically meaningful thresholds before routine clinical use.

## 6 Future directions

Future studies should recruit larger, multi-site, demographically balanced normative and aphasia samples across Saudi regions and other Arabic-speaking communities, with planned stratification by age, education, gender, and regional dialect, and should evaluate item-level name agreement and psycholinguistic characteristics such as lexical frequency, imageability, age of acquisition, and visual complexity (18). This future-validation agenda is consistent with broader calls for cross-linguistically informed and linguistically fair neurogenic-language assessment in understudied language communities (16, 17, 23). These studies should use prospectively specified scoring procedures and a detailed scoring manual that documents accepted regional variants and item-specific decisions. Future work should also include formal inter-rater and test–retest reliability analyses, item-level psychometric evaluation, and independent assessment of diagnostic accuracy and clinically meaningful thresholds. Testing in acoustically controlled environments with head-mounted microphones, standardized stopping rules or rest breaks, and consistent recording procedures may improve response-time precision and scoring consistency. Ambiguous stimulus items should be re-illustrated or replaced where indicated by item-level findings.

## Data Availability

The raw data supporting the conclusions of this article will be made available by the authors, without undue reservation.
